# MiR-30e-5p and MiR-15a-5p Expressions in Plasma and Urine of Type 1 Diabetic Patients With Diabetic Kidney Disease

**DOI:** 10.3389/fgene.2019.00563

**Published:** 2019-06-12

**Authors:** Cristine Dieter, Taís Silveira Assmann, Aline Rodrigues Costa, Luís Henrique Canani, Bianca Marmontel de Souza, Andrea Carla Bauer, Daisy Crispim

**Affiliations:** ^1^Endocrine Division, Hospital de Clínicas de Porto Alegre, Porto Alegre, Brazil; ^2^Post-Graduate Program in Medical Sciences: Endocrinology, Faculdade de Medicina, Universidade Federal do Rio Grande do Sul, Porto Alegre, Brazil; ^3^Department of Food Science and Physiology, School of Pharmacy and Nutrition, University of Navarra, Pamplona, Spain; ^4^Nephrology Division, Hospital de Clínicas de Porto Alegre, Porto Alegre, Brazil

**Keywords:** microRNA expression, miR-15a-5p, miR-30e-5p, diabetic kidney disease, bioinformatics analysis, type 1 diabetes mellitus

## Abstract

**Introduction:**

Diabetic kidney disease (DKD) is a common microvascular complication that affects 40% of patients with diabetes mellitus (DM). Emerging evidence suggests a role for several microRNAs (miRNAs) in the development of DKD. In this context, miR-15a-5p and miR-30e-5p have been shown to regulate the expression of the uncoupling protein 2 (UCP2), a mitochondrial protein that decreases reactive oxygen species (ROS) formation by the mitochondria. Since ROS overproduction is a key contributor to the pathogenesis of DKD, dysregulation of these two miRNAs could be involved in DKD pathogenesis. Thus, the aim of this study was to compare the expressions of miR-15a-5p and miR-30e-5p in type 1 DM (T1DM) patients with DKD (cases) and without this complication (controls), and to perform bioinformatics analyses to investigate their putative targets and biological pathways under their regulation.

**Methods:**

MiR-15a-5p and miR-30e-5p expressions were analyzed in plasma and urine of 17 T1DM controls and 23 DKD cases (12 with moderate DKD and 11 with severe DKD) using qPCR. Bioinformatics analyses were performed in Cytoscape software.

**Results:**

MiR-30e-5p expression was downregulated in plasma of patients with moderate and severe DKD compared to T1DM controls. Moreover, this miRNA was also downregulated in urine of patients with severe DKD compared to the other groups. No difference was found in miR-15a-5p expression between groups. Bioinformatics analyses indicated that miR-30e-5p and miR-15a-5p regulate various genes that participate in pathways related to angiogenesis, apoptosis, cell differentiation, oxidative stress, and hypoxia.

**Conclusion:**

MiR-30e-5p seems to be downregulated in plasma and urine of patients with DKD.

## Introduction

Diabetic kidney disease (DKD) is a common microvascular complication that occurs in approximately 40% of patients with diabetes mellitus (DM), and that can lead to end-stage renal disease (ESRD) (Gross et al., 2005). DKD is clinically characterized by albuminuria and/or a gradual reduction in the glomerular filtration rate (GFR) (Ritz et al., 2011). Pathological changes in renal cells of DKD patients include glomerular hypertrophy, mesangial expansion, and tubulointerstitial fibrosis due to the accumulation of extracellular matrix (ECM) proteins, thickening of basement membrane, and podocyte dysfunction (Assmann et al., 2018b). At the cellular level, chronic hyperglycemia causes reactive oxygen species (ROS) overproduction by the mitochondria, which then triggers key pathways related to DKD: increased formation of advanced glycation end-products (AGEs) and overexpression of their receptors (RAGEs); activation of protein kinase C isoforms; increased flux of glucose by the polyol pathway; and upregulation of the hexosamine pathway (Brownlee, 2005; Giacco and Brownlee, 2010; Thomas et al., 2015).

The main risk factors for DKD are duration of chronic hyperglycemia, arterial hypertension, dyslipidemia, and genetic and epigenetic components (Brennan et al., 2013; Assmann et al., 2018b). Regarding epigenetic factors, emerging evidence has suggested an important role of microRNAs (miRNAs) in the pathogenesis of DKD (Kato and Natarajan, 2015; Wanner and Bechtel-Walz, 2017; Assmann et al., 2018a,b). MiRNAs are small (≅21–23 nucleotides) non-coding RNAs that regulate gene expression of 60% of protein coding genes (Assmann et al., 2017); thus, regulating many cellular functions and influencing the development and progression of a number of diseases (Kato and Natarajan, 2015; Assmann et al., 2017, 2018b).

In this context, miR-15a-5p and miR-30e-5p target the uncoupling protein 2 (*UCP2*) gene (Sun et al., 2011; Jiang et al., 2013). UCP2 is a mitochondrial protein that seems to mildly uncouples the oxidative phosphorylation from ATP synthesis by dissipating the proton gradient generated across the mitochondrial inner membrane, consequently decreasing ATP production and reducing ROS formation by the mitochondria (Brand and Esteves, 2005; Fisler and Warden, 2006; Souza et al., 2011). Taking into account the role of UCP2 in decreasing oxidative stress, and that *UCP2* gene polymorphisms have been associated with DKD and other diabetic complications (Rudofsky et al., 2006; Tiwari et al., 2009; Crispim et al., 2010; Souza et al., 2011; de Souza et al., 2015), dysregulation of miR-15a-5p and miR-30e-5p might also be involved in DKD pathogenesis.

Accordingly, experimental studies have linked both miRNAs to podocyte injury, epithelial-mesenchymal transition (EMT) in tubular epithelial cells, and kidney fibrosis, which are features related to chronic kidney disease (CKD) and DKD (Jiang et al., 2013; Sun et al., 2014; Wu et al., 2014, 2015; Guo et al., 2017; Zhao D. et al., 2017). In humans, miR-15a-5p was reported as being downregulated in urine of patients with CKD or DKD compared to healthy controls (Khurana et al., 2017; Xie et al., 2017). Expression of miR-30e-5p was also downregulated in urinary exosomes of DKD patients compared to healthy subjects or type 2 DM patients without this complication (Delic et al., 2016). Moreover, expression of this miRNA in urine was correlated with proteinuria levels in DKD patients (Cardenas-Gonzalez et al., 2017). Even though these studies have associated dysregulation of miR15a-5p and miR-30e-5p with DKD, their exact roles and clinical relevance remain unknown. Therefore, in the present study, we analyzed miR-15a-5p and miR-30e-5p expressions in plasma and urine of type 1 DM (T1DM) patients with and without DKD. Moreover, we carried out bioinformatics analyses to investigate the putative targets and biological pathways under regulation of these two miRNAs.

## Materials and Methods

### Sample and Phenotype Measurements

This case-control study was designed following STROBE guidelines for reporting of association studies (von Elm et al., 2014). The sample comprised 40 T1DM patients, who were divided in 17 patients without DKD (control group) and 23 DKD cases (12 with moderate DKD and 11 with severe DKD). All T1DM patients included in the study were from outpatient clinics at Hospital de Clínicas de Porto Alegre or Instituto da Criança com Diabetes at Grupo Hospitalar Conceição (Rio Grande do Sul, Brazil), and were recruited between August 2014 and July 2018, accordingly to the flowchart showed in the Supplementary Figure 1. T1DM diagnosis was based on the American Diabetes Association criteria (American Diabetes Association, 2018).

Diabetic kidney disease was classified based on the Kidney Disease Improving Global Outcomes (KDIGO) guidelines (Andrassy, 2013). T1DM patients were divided into 3 groups according to their renal function: (1) patients with ≥10 years of T1DM and without DKD [urinary albumin excretion (UAE) <30 mg/g and estimated GFR (eGFR) ≥60 ml/min/1.73 m^2^; T1DM control]; (2) patients with moderate DKD (UAE <30 and eGFR 30–59 or UAE 30–300 and eGFR ≥45 or UAE >300 and eGFR >60); and (3) patients with severe DKD (UAE <30 and eGFR <29 or UAE 30–300 and eGFR <44 or UAE >300 and eGFR <59). Exclusion criteria were any febrile illness during the last 3 months, chronic inflammatory or rheumatic diseases, hepatitis, HIV-positivity, glucocorticoid treatment, liver or cardiac failure, kidney transplantation, hereditary dyslipidemia, and inborn or acquired errors of metabolism excepting DM. In order to avoid bias, we selected this extensive list of exclusion criteria since they might interfere with miRNA expression. Moreover, all samples were collected during the morning, since the period of the day might also influence miRNA expression.

A standard questionnaire was used to collect information on age, age at diagnosis, T1DM duration, drug treatment, and ethnicity. The ethnic group was defined based on self-classification. All patients underwent physical and laboratory evaluations, as previous described (Assmann et al., 2014). Serum creatinine was measured by the Jaffé reaction and UAE by immunoturbidimetry (Sera- Pak immuno microalbuminuria, Bayer, Tarrytown, NY, United States) (Zelmanovitz et al., 1997). Estimated GFR was calculated using the Chronic Kidney Disease Epidemiology Collaboration (CKD-EPI) equation: eGFR = 141 × min (SCR/κ, 1)^α^ × max (SCR/κ, 1)^-1,209^ × 0,993^*age*^ × 1,018 (if female) × 1,159 (if black) (Levey et al., 2009). All subjects gave written informed consent in accordance with the Declaration of Helsinki. The study protocol was approved by the Ethic Committees in Research from Hospital de Clínicas de Porto Alegre and Grupo Hospitalar Conceição/Instituto da Criança com Diabetes.

### RNA Extraction and Quantification of miRNA Expressions by qPCR

Peripheral blood samples of all subjects were collected in the morning with at least 8 h of fasting, in EDTA-coated tubes. Midstream 20 ml voided urine samples were also collected from all patients. Immediately after collection, blood and urine samples were centrifuged at 3500 rpm for 15 min at 4°C, and their aliquots were stored at -80°C until quantification of miRNA expressions. Total RNA was isolated from 450 μl of plasma or urine using the MiRVana PARIS miRNA Isolation Kit (Ambion, Thermo Fisher Scientific, DE, United States), according to the manufacturer’s instructions. Purity and concentration of RNA samples were measured using the NanoDrop ND-1000 Spectrophotometer (Thermo Fisher Scientific). Only RNA samples that achieved adequate purity ratios (A260/A280 = 1.9–2.1) were used for subsequent analyses (Bustin et al., 2009).

Real-time quantitative PCR (qPCR) was performed in two separate reactions: first, the total RNA was reverse-transcribed into cDNA and, second, the cDNA was amplified by qPCR. Reverse transcription of 2 ng/μl of RNA into cDNA was carried out using TaqMan miRNA RT assays (Thermo Fisher Scientific) specific for each miRNA of interest (assay reference numbers: 000389 for hsa-miR-15a-5p, and 002223 for hsa-miR-30e-5p). The *small nuclear RNA U6* (*U6snRNA*) gene was used as the reference gene (assay reference number: 001973).

Next, qPCR experiments were carried out in a ViiA^TM^ 7 Fast Real-Time PCR System. PCR reactions were performed using 0.5 μl TaqMan miRNA Assays 20× (Thermo Fisher Scientific) for target miRNAs or *U6snRNA*, 5 μl TaqMan Universal PCR Master Mix II no UNG 2×, and 1 μl of cDNA (10 ng/μl), in a total volume of 10 μl. Each sample was assayed in triplicate and a negative control (without any cDNA) was included in each experiment. Cycling conditions for these genes were an initial cycle of 95°C for 10 min, followed by 50 cycles of 95°C for 15 s and 60°C for 90 s. Quantification of the two target miRNAs was performed using the 2^-ΔΔCq^ method and the *U6snRNA* gene as the reference and are shown as n-fold changes in relation to the calibrator sample (Bustin et al., 2009). The calibrator sample was a pool of cDNAs from the samples used in the study.

### Bioinformatics Analyses

To better understand the functional involvement of miR-15a-5p and miR-30e-5p in DKD, these miRNAs were submitted to bioinformatics analyses to investigate their putative target genes and find possible biological pathways under their regulation. Bioinformatics analyses were performed using the Cytoscape v. 3.2.1 software (Shannon et al., 2003) with two plugin tools: (1) CyTargetLinker (Kutmon et al., 2013), and (2) Biological Networks Gene Ontology (BiNGO) (Maere et al., 2005).

The CyTargetLinker v3.0.1 was used to search for validated and predicted miRNA-target gene interactions (MTI) and visualize them in a graphical way. For this study, we obtained Homo sapiens MTIs from one experimentally validated database (miRTarBase v.4.4) and from two predicted miRNA databases (MicroCosm v.5.0 and TargetScan v.6.2).

Next, functional enrichment analysis of miRNA-target genes was performed to retrieve gene ontology (GO) annotations for miR-15a-5p and miR-30e-5p target genes that were identified with the CyTargetLinker, using the BiNGO plug-in in the Cytoscape environment. This investigation was performed for targets of each individual miRNA as well as for targets of the two miRNAs analyzed together. Significance for GO pathway enrichment was estimated with a hypergeometric test and adjusted for multiple hypotheses using the Benjamini-Hochberg and Hochberg False Discovery Rate (FDR) test. Pathways with a *q*-value <0.05 were considered strongly enriched for the genes targeted by the two miRNAs analyzed.

### Statistical Analyses

Normal distribution of variables was checked using the Kolmogorov Smirnov and Shapiro–Wilk tests. Variables with normal distribution are presented as mean ± SD. Variables with skewed distribution were log-transformed before analyses and are presented as median (25–75th percentiles). Categorical data are shown as percentages. Clinical and laboratory characteristics were compared among groups using One-way ANOVA tests or χ^2^-tests, as appropriate. MiRNA expressions were compared between groups using Kruskal–Wallis tests. Correlations between quantitative variables were analyzed using Spearman’s correlation tests. All statistical analyses were performed using the SPSS statistical package (v.18.0) for Windows (SPSS Inc., Chicago, IL, United States), and *P*-values <0.05 were considered statistically significant.

The sample size was calculated in the OpenEpi site^1^ and based on previous studies (Cardenas-Gonzalez et al., 2017; Xie et al., 2017). Considering a power of 80% (α = 0.05) to detect two fold changes (±1.5 SD) in miRNA expressions between case and control groups, we needed at least 10 patients in each group in order to have an adequate statistical power.

## Results

### Characteristics of the T1DM Patients

Clinical and laboratorial characteristics of the T1DM control patients and DKD cases included in this study are summarized in Table 1. Gender, ethnicity, BMI, age at T1DM diagnosis, HDL cholesterol, and total cholesterol levels did not differ between cases with moderate or severe DKD and T1DM controls. HbA1c levels were higher in both moderate and severe DKD groups compared to controls (*P* = 0.005). As expected, age, T1DM duration, prevalence of hypertension and diabetic retinopathy were higher in severe DKD patients compared to patients with moderate DKD and T1DM controls (*P* < 0.050) (Table 1).

**Table 1:** Clinical and laboratory characteristics of T1DM controls and DKD cases.

Characteristics	T1DM controls (*n* = 17)	Moderate DKD (*n* = 12)	Severe DKD (*n* = 11)	*P*^∗^
Age (years)^†^	24.2 ± 5.5^a^	21.8 ± 4.1^a^	30.6 ± 5.7^b^	0.001
Gender (% male)	52.9	54.5	36.4	0.624
Ethnicity (% black)	5.9	18.2	9.1	0.571
BMI (kg/m^2^)	23.2 ± 3.3	22.6 ± 1.9	23.7 ± 3.8	0.738
HbA1c (%)^†^	8.6 ± 0.9^a^	10.5 ± 2.1^b^	10.2 ± 1.5^b^	0.005
Hypertension (%)^†^	11.8^a^	9.1^a^	70.0^b^	0.001
Age at diagnosis (years)	9.0 (2.0–12.5)	5.0 (3.0–8.0)	6.0 (6.0–8.0)	0.812
Duration of diabetes (years)^†^	15.6 ± 5.0^a^	15.3 ± 5.8^a^	23.7 ± 5.2^b^	<0.0001
Total cholesterol (mg/dL)	168.3 ± 34.4	189.4 ± 78.6	183.6 ± 46.4	0.678
Triglycerides (mg/dL)	69.0 (45.0–107.0)	126.5 (67.0–157.5)	113.5 (65.7–138.2)	0.096
HDL Cholesterol (mg/dL)	46.5 ± 11.9	60.6 ± 17.7	58.7 ± 22.8	0.187
Creatinine (μg/dl)	0.7 (0.6–0.9)	0.9 (0.8–1.2)	4.5 (1.0–8.1)	–
eGFR (mL/min per 1.73 m2)	123.0 (112.5–126.0)	112.0 (87.7–127.7)	16.0 (6.0–87.5)	–
UAE (mg/g)	6.1 (3.3–9.3)	76.9 (36.0–168.7)	740.7 (410.3–2551.8)	–
Diabetic retinopathy (%)^†^	5.9^a^	11.1^a^	70.0^b^	0.001


*Variables are shown as mean ± SD, median (25–75th percentiles) or %. ^∗^*P*-values for age, BMI, HbA1c, age at diagnosis, duration of diabetes, cholesterol total, triglycerides, and HDL cholesterol were computed using One-way ANOVA. *P*-values for gender, ethnicity, hypertension, and diabetic retinopathy were computed using χ^*2*^-tests. ^†^Equal letters mean equal values and different letters mean different values. BMI, body mass index; DKD, diabetic kidney disease; eGFR, estimated glomerular filtration rate; HbA1c, glycated hemoglobin; T1DM, type 1 diabetes mellitus; UAE, urinary albumin excretion.*

### Expressions of MiR-15a-5p and MiR-30e-5p in Plasma and Urine of T1DM Patients With or Without DKD

**FIGURE 1:**
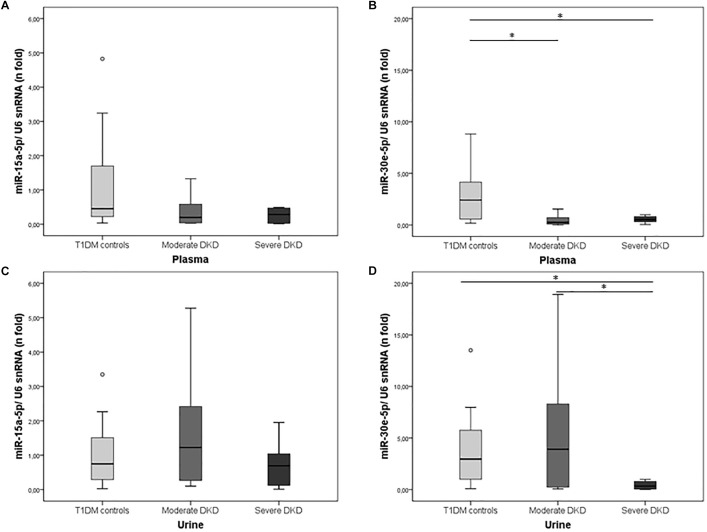
Expression of miR-15a-5p and miR-30e-5p in plasma and urine of T1DM controls and cases with moderate or severe DKD. Relative expressions of **(A)** miR-15-5p and **(B)** miR-30e-5p in plasma, and **(C)** miR-15-5p and **(D)** miR-30e-5p in urine samples of T1DM controls and patients with moderate or severe DKD. Expressions were evaluated using qPCR. Results are expressed as n-fold changes in relation to the calibrator sample (ΔΔCq method), using the *U6 snRNA* as the reference gene, and are shown as median (25–75th percentiles). *P*-values were obtained using Kruskal–Wallis tests. ^∗^*P* < 0.050.

Expressions of miR-15a-5p and miR-30e-5p were investigated in plasma and urine of T1DM controls and DKD cases grouped according to the severity of this complication. In plasma, miR-30e-5p expression was downregulated in both severe and moderate DKD patients compared to T1DM controls [severe DKD: 0.53 (0.25–0.84), moderate DKD: 0.25 (0.08–0.82), and T1DM controls: 2.42 (0.51–4.33), *P* = 0.003; Figure 1B]. In urine samples, miR-30e-5p expression was only downregulated in the severe DKD group compared to moderate DKD and T1DM control groups [severe DKD: 0.34 (0.05–0.85), moderate DKD: 3.92 (0.23–9.66), and T1DM controls: 2.96 (0.99–5.97), *P* = 0.017; Figure 1D]. MiR-15a-5p expression in plasma and urine did not differ between groups (*P* > 0.050; Figure 1A,C).

We next evaluated possible correlations between miR-15a-5p and miR-30e-5p expressions in plasma and DKD-related measurements (eGFR, creatinine, and UAE levels) and HbA1c. MiR-15a-5p and miR-30e-5p expressions showed significant negative correlations with UAE levels (*r* = -0.459, *P* = 0.016 and *r* = -0.617, *P* = 0.0001, respectively) and HbA1c levels (*r* = -0.432, *P* = 0.009 and *r* = -0.435, *P* = 0.004, respectively). No significant correlation was found between the two analyzed miRNAs and eGFR values or creatinine levels.

### Target Prediction and Functional Enrichment Analysis for MiR-15a-5p and MiR-30e-5p

**FIGURE 2:**
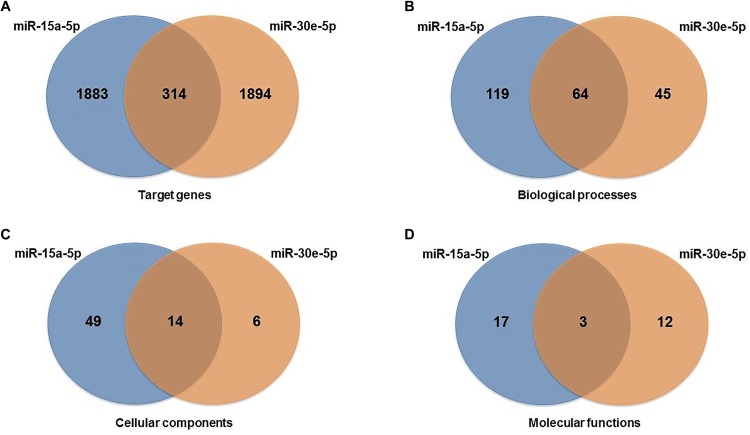
Venn diagrams showing interactions between miR-15a-5p and miR-30e-5p and their target genes and gene ontology pathways: **(A)** target genes, **(B)** biological process pathways, **(C)** cellular component pathways, and **(D)** molecular function pathways shared between the two miRNAs analyzed.

**FIGURE 3:**
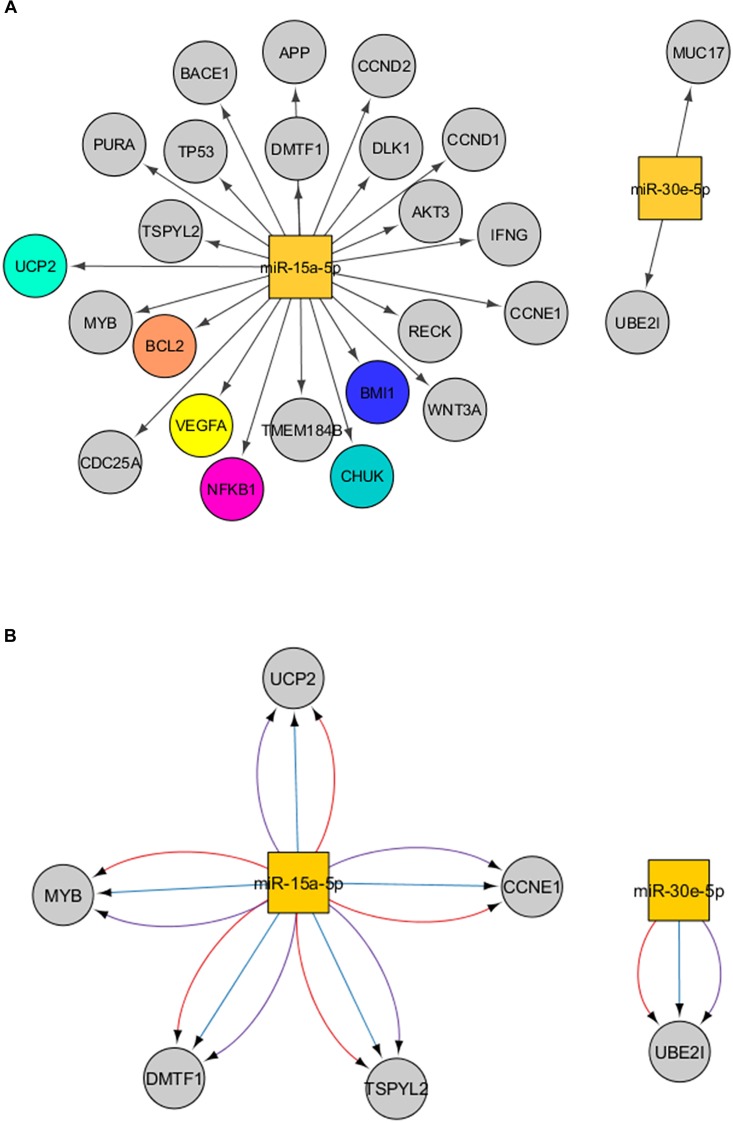
Interactions between miR-15a-5p and miR-30e-5p and their target genes. **(A)** Experimentally validated target genes of the miRNAs analyzed. Colored genes are those previously associated with DKD. **(B)** Significant target genes of the miRNAs analyzed found in the three databases analyzed (validated + computationally predicted). Lines in red mean interactions retrieved from miRTarBase, in blue from microcosm, and in purple from TargetScan. Squares represent miRNAs and the circles represent their target genes.

Target prediction of the miR-15a-5p and miR-30e-5p was performed using distinct bioinformatics resources in the Cytoscape environment. Using the strategy described in the Material and Methods Section, 2197 genes were identified as putative targets of the miR-15a-5p, while 2208 genes were identified as putative targets of the miR-30e-5p. Of note, 314 targets were modulated by both miRNAs (Figure 2A and Supplementary Figure 2). After that, we analyzed only the experimentally validated target genes of these two miRNAs (Figure 3). As shown in Figure 3A, 23 validated target genes were found for miR-15a-5p and only two validated targets for miR-30e-5p. Among the validated target genes found for miR-15a-5p, some of them have been reported as being associated with kidney dysfunction or DKD pathogenesis, including *BCL2, VEGFA, UCP2, BMI1*, and *NFKB1* and its inhibitor *CHUK* (*IKKA*) (Figure 3A). Figure 3B shows those targets of miR-15a-5p and miR-30e-5p that were found in all 3 databases analyzed of MITs (one database of experimentally validated targets and two of computationally predicted targets). As can be observed in this figure, *UCP2* is a predicted and validated target of miR-15a-5p, being present in all the 3 analyzed databases (Figure 3B). Of note, most validated MTIs shown in Figure 3A were not computationally predicted (Figure 3B), demonstrating the importance of using distinct databases for target gene analysis.

To explore the biological pathways possibly affected by the two miRNAs analyzed, we carried out functional enrichment analysis of their target genes using pathways maps from the BiNGO Database. GO pathways were investigated for biological, cellular, and molecular processes associated with the set of predicted and validated target genes found for miR-15a-5p and miR-30e-5p in the previous analysis. A total of 250 unique significant pathways were enriched for miR-15a-5p, being 183 pathways involved in biological processes, 63 in cellular components, and 20 in molecular functions (Supplementary Table 1). Of note, some of these pathways participate in more than one biological category of BiNGO. For miR-30e-5p, a total of 142 unique significant pathways were enriched, being 109 pathways involved in biological processes, 20 in cellular components, and 15 in molecular functions (Supplementary Table 2).

**FIGURE 4:**
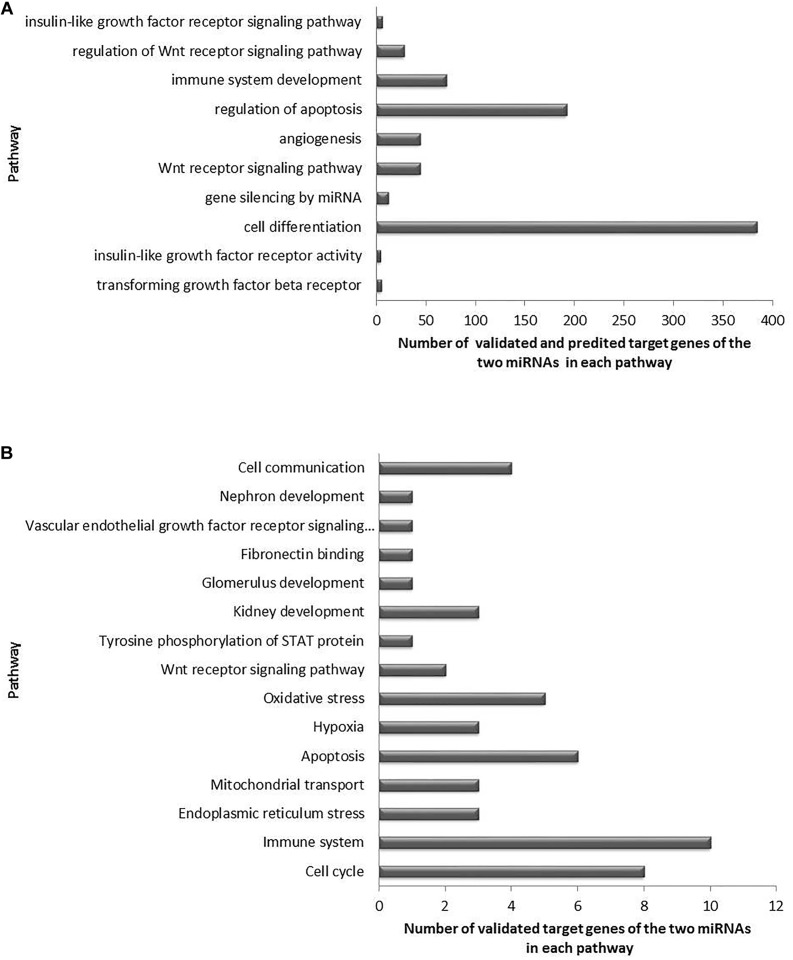
Significant enriched pathways related to DKD pathogenesis and regulated by the target genes of miR-15a-5p and miR-30e-5p. **(A)** Top 10 selected pathways of predicted and validated target genes of both miRNAs, and **(B)** selected pathways of validated target genes of both miRNAs.

Moreover, a total of 81 unique pathways were enriched for both miRNAs, being 64 pathways involved in biological process (Figure 2B), 14 in cellular components (Figure 2C), and 3 in molecular functions (Figure 2D). Many of these pathways are well known to be related to DKD pathogenesis, such as transforming growth factor beta receptor, oxidative stress, apoptosis, VEGF and angiogenesis, endoplasmic reticulum stress, hypoxia, and mitochondrial transport pathways (Figure 4A for pathways derived from predicted + validated targets, and Figure 4B for pathways derived only from validated targets).

## Discussion

Chronic hyperglycemia may induce cellular damage through increased production of ROS, which then seems to trigger the main pathways related to microvascular diabetic complications, including DKD (Giacco and Brownlee, 2010). Recent studies have shown that miR-15a-5p and miR-30e-5p are associated with podocyte injury, EMT in tubular epithelial cells, and kidney fibrosis (Jiang et al., 2013; Sun et al., 2014; Wu et al., 2014, 2015; Guo et al., 2017; Zhao D. et al., 2017), and also seem to be dysregulated in urine of patients with DKD or CKD (Delic et al., 2016; Cardenas-Gonzalez et al., 2017; Khurana et al., 2017; Xie et al., 2017). Interestingly, both miRNAs target *UCP2* (Sun et al., 2011; Jiang et al., 2013), a mitochondrial protein that seems to have a key role in decreasing oxidative stress (Souza et al., 2011). Based on these findings, we therefore analyzed miR-15a-5p and miR-30e-5p expressions in T1DM patients according to DKD stages. Our results indicate that miR-30e-5p is downregulated in plasma and urine of T1DM patients with DKD compared to patients without this complication. No difference was found in the miR-15a-5p expressions between groups.

In agreement with our results, miR-30e-5p expression was also downregulated in urinary exosomes of type 2 DM patients with DKD compared to healthy controls or diabetic patients without this complication (Delic et al., 2016). This association is biologically plausible since all miR-30 family members seem to be essential for structural and functional homeostasis of podocytes, where they are abundantly expressed (Shi et al., 2013; Wu et al., 2014, 2015; Guo et al., 2017). TGF-β1 treatment downregulated miR-30 expression in glomerular podocytes *in vivo* and *in vitro*, and the sustained expression of miR-30 inhibited TGF-β1-induced apoptosis of podocytes, while its knockdown aggravated podocyte injury (Shi et al., 2013; Wu et al., 2014). Wu et al. (2014) showed that miR-30 exerts their protective roles by direct inhibition of *Notch1* and *p53*, which mediate podocyte injury. Moreover, miR-30 inhibits the excessive activation of calcium/calcineurin signaling, preventing cytoskeletal damage and apoptosis of podocytes (Wu et al., 2015; Zhao Y. et al., 2017). Zhao D. et al. (2017) reported that in renal tissue of over-8-week-old db/db mice and in human renal tubular epithelial cells (RTECs) cultured for 6 h in high glucose (HG), miR-30e-5p was downregulated while its target *GLIPR-2*, involved in EMT, was upregulated. Besides, miR-30e-5p overexpression in RTECs promoted proliferation of these cells and inhibited EMT, which could avoid renal fibrosis in DKD (Zhao D. et al., 2017). Accordingly, Jiang et al. (2013) showed that miR-30e-5p was also downregulated in RTECs from mice with ureteral occlusion-induced kidney fibrosis and in TGF-β1-treated NRK-52E renal cells. Transfection of a miR-30e-5p mimic in NRK-52E cells reduced TGF-β1-induced *UCP2* expression, inhibiting EMT, whereas a miR-30e inhibitor promoted epithelial cell phenotype changes by loss of E-cadherin, induction of α-SMA, and fibrinogen expression (Jiang et al., 2013). Thus, the downregulation of miR-30e-5p has consistently been shown to be involved in renal fibrosis pathogenesis. Although here we observed that miR-30e-5p was downregulated in plasma of DKD patients independently of the disease severity; in urine, this miRNA was only downregulated in patients with severe DKD, suggesting that it may be a marker of disease progression.

Our bioinformatics analyses indicated that *UBE2I* (ubiquitin-conjugating enzyme E2 I) and *MUC17* (mucin 17) genes are validated target genes of miR-30e-5p (Figure 3A). UBE2I, also known as UCB9, constitutes a core machinery in the sumoylation pathway. Sumoylation is a process in which a small ubiquitin-like modifier (SUMO) is covalently attached to other proteins, modifying their functions (Neyret-Kahn et al., 2013). Important roles for sumoylation were shown in heterochromatin configuration, and sumoylation of core histones negatively regulates transcription (Neyret-Kahn et al., 2013). MiR-30e-5p-induced downregulation of *UBE2I* inhibited the proliferation and migration of vascular smooth muscle cells (Zong et al., 2017). MUC17 is a glycoprotein characterized as a membrane-bound mucin that provides protection to gut epithelial cells (Gum et al., 2002; Luu et al., 2010). Although UBE2I and MUC17 have not been studied in the context of kidney dysfunction or DKD, a polymorphism in the *MUC17* gene was previously associated with protection for CKD in an exome-wide association study (Yamada et al., 2018).

MiR-15a-5p regulates several genes involved in cell division, metabolism, stress response, apoptosis, and angiogenesis (Finnerty et al., 2010). It is abundantly expressed in human and mouse renal tissue (Finnerty et al., 2010), although only few studies have evaluated its function regarding CKD and DKD. In contrast with our results, miR-15a-5p expression was decreased in urinary exosomes of patients with DKD or CKD compared to controls (Khurana et al., 2017; Xie et al., 2017). In addition, this miRNA was downregulated by HG in RTECs (Sun et al., 2014). Treatment of RTECs with a miR-15a-5p mimic was able to reverse HG-induced EMT in these cells, since it inhibited *α-SMA* and *collagen I* expressions, and restored *E-cadherin* expression (Sun et al., 2014). Although our study did not demonstrate an association between miR-15a-5p expression in plasma and urine of patients with DKD, we observed a negative correlation with UAE levels, suggesting an undescribed role of miR-15a-5p in the glomerular basement membrane integrity. MiR-15a-5p was also negatively correlated with HbA1c levels, which is in accordance with the study by Flowers et al. (2015) that reported a negative correlation between circulating miR-15a-5p expression and blood glucose levels in Asian Indians who had glycemic increment after 2.5 years of follow up compared to those who remained stable.

Our bioinformatics analysis using only an experimentally validated database showed that miR-15a-5p regulates genes from several pathways involved in kidney dysfunction mechanisms and DKD development (Kanwar et al., 2011): *VEGFA, BCL2, NFKB1* and its inhibitor *CHUK* (*IKKA*), *UCP2*, and *BMI1* (Figure 3A). When we used both experimentally validated and computationally predicted tools, *UCP2, CCNE1, TSPYL2, DMTF1*, and *MYB* remained as significant targets of miR-15a-5p (Figure 3B). To date, only *UCP2* and *TSPYL2* genes have been previously investigated regarding kidney dysfunction.

As already mentioned, UCP2 seems to decrease oxidative stress, being a candidate gene for DKD. Sun et al. (2011) confirmed experimentally in MIN-6 cells that miR-15a-5p directly targets *UCP2*, decreasing mitochondrial uncoupling and, thus, increasing insulin biosynthesis in this beta-cell line since UCP2 is a negative regulator of insulin secretion. Regarding kidney dysfunction, Qiu et al. (2012) reported that oral administration of genipin (a UCP2 inhibitor) partially prevented the progression of DKD in C57BL/6J mice by improving podocyte function. Accordingly, UCP2 was induced in RTECs after unilateral ureteral obstruction in mice, while those mice with ablated *UCP2* resisted obstruction-induced EMT and kidney fibrosis (Jiang et al., 2013). Additionally, *UCP2* knockdown in NRK-52E tubular cells abolished the effect of TGF-β1 treatment, decreasing ECM production (Jiang et al., 2013). In contrast, Chen et al. (2014) showed that inhibition of UCP2 by genipin increased oxidative stress in rat RTECs treated with HG medium, leading to increased apoptosis. *UCP2* knockdown in renal mesangial cells of rats also increased oxidative stress, inflammation and apoptosis *in vitro* (Di Castro et al., 2013). Therefore, it is still not clear if UCP2 has a protective or deleterious effect on renal function.

Regarding *TSPYL2* (testis-specific protein Y-encoded like 2), also known as CDA1, it acts in chromatin remodeling and as inhibitor of cell proliferation in response to DNA damage (Tao et al., 2011). Interestingly TSPYL2 is a regulator of cell-cycle arrest induced by TGF-β1 (Epping et al., 2015), which is a major player in DKD pathogenesis mainly because of its potent pro-fibrotic actions (Assmann et al., 2018b). Accordingly, *TSPYL2* expression was upregulated in the aorta of a murine diabetic model of atherosclerosis (Pham et al., 2010). *In vitro* studies in vascular cells showed that TGF-β1 treatment increased TSPYL2 protein, which then amplified TGF-β1 signaling leading to upregulation of ECM genes (Pham et al., 2010). Chai et al. (2013) reported that *TSPYL2* knockout in diabetic mice reduced expression of TGF-β1 receptors in the kidney as well as reduced renal matrix accumulation and attenuated glomerular and tubulointerstitial injury. Therefore, this gene might be a new candidate gene for DKD.

This study has a few limitations. First, we cannot exclude the possibility of type II error when comparing expressions of the two analyzed miRNAs between groups. However, this bias was minimized since our sample size has a power of 80% (α = 0.05) to detect two fold changes (±1.5 SD) in miRNA expressions between case and control groups. Second, duration of T1DM was significantly higher in patients with severe DKD compared to the other two groups, which was expected since diabetes duration is associated with increased prevalence of diabetic chronic complications (Gross et al., 2005). Although this bias could have influenced our results, it is a conservative bias since if some control patients have already some predisposing factor for later development of DKD, this would only decrease the observed association. Third, many factors, such as presence of inflammatory diseases as well as use of medications, can interfere on miRNA expression. In order to reduce this possibility, we used an extensive list of exclusion criteria, as described in the Material and Methods Section. Lastly, as already mentioned many studies have suggested that hyperglycemia induces oxidative stress, which then triggers the main pathways related to diabetic chronic complications; however, few recent studies have challenged the increased mitochondrial ROS production in diabetic kidney disease (Dugan et al., 2013; Sharma, 2015; Coughlan and Sharma, 2016). The reduction of ROS production by UCP2 is also a matter of discussion that needs further investigation (Couplan et al., 2002; Nedergaard and Cannon, 2003). Even though these controversial premises, our main result showing that miR-30e-5p expression is downregulated in patients with DKD is not necessarily influenced by this limitation since this miRNA has many other targets, including TGF-β1. Thus, despite these limitations, our present data is important to be reported since this is the first study that evaluated miR-30e-5p and miR-15a-5p in both plasma and urine samples and in a Brazilian population.

## Conclusion

We demonstrated that miR-30e-5p is downregulated in plasma and urine of DKD patients. MiR-15e-5p was only negatively correlated with UAE levels in T1DM patients. Bioinformatics analyses suggest that both analyzed miRNAs regulate genes involved in key mechanisms related to DKD pathogenesis, such as TGF-β receptor, angiogenesis, apoptosis, and hypoxia. Moreover, they are involved in oxidative stress pathway (probably by targeting *UCP2*), which is an important mechanism linking hyperglycemia to diabetic chronic complications. Our study also suggests that *CCNE1, DMTF1, TSPYL2, MYB* and *UBE2I* might constitute new potential candidate genes for DKD.

## Ethics Statement

This study was carried out in accordance with the recommendations of the Ethic Committees in Research from Hospital de Clínicas de Porto Alegre and Grupo Hospitalar Conceição/Instituto da Criança com Diabetes with written informed consent from all subjects. All subjects gave written informed consent in accordance with the Declaration of Helsinki. The protocol was approved by the Ethic Committees in Research from Hospital de Clínicas de Porto Alegre and Grupo Hospitalar Conceição/Instituto da Criança com Diabetes.

## Author Contributions

CD designed the study, researched the data, collected the samples, performed the experiments, and wrote the manuscript. AC collected the samples, researched the data, and reviewed the manuscript. TA researched the data, collected the samples, performed the bioinformatics analyses, contributed to discussion, and reviewed the manuscript. AB, BdS, and LC contributed to discussion and reviewed the manuscript. DC designed the study, contributed to the discussion, and wrote the manuscript.

## Conflict of Interest Statement

The authors declare that the research was conducted in the absence of any commercial or financial relationships that could be construed as a potential conflict of interest.

## Footnotes

1 www.openepi.com
