# Supplementary figures and images for: MiR-30e-5p and MiR-15a-5p Expressions in Plasma and Urine of Type 1 Diabetic Patients With Diabetic Kidney Disease
Source: Front Genet. 2019 Jun 12;10:563. doi: 10.3389/fgene.2019.00563 (PMC6582252; doi:10.3389/fgene.2019.00563)

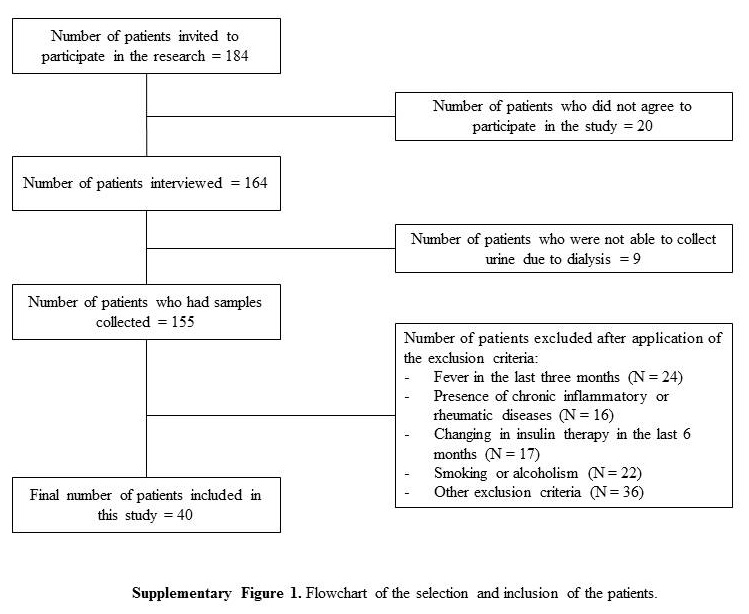

Supplement: Supplementary file 1 [file Image_1.JPEG]

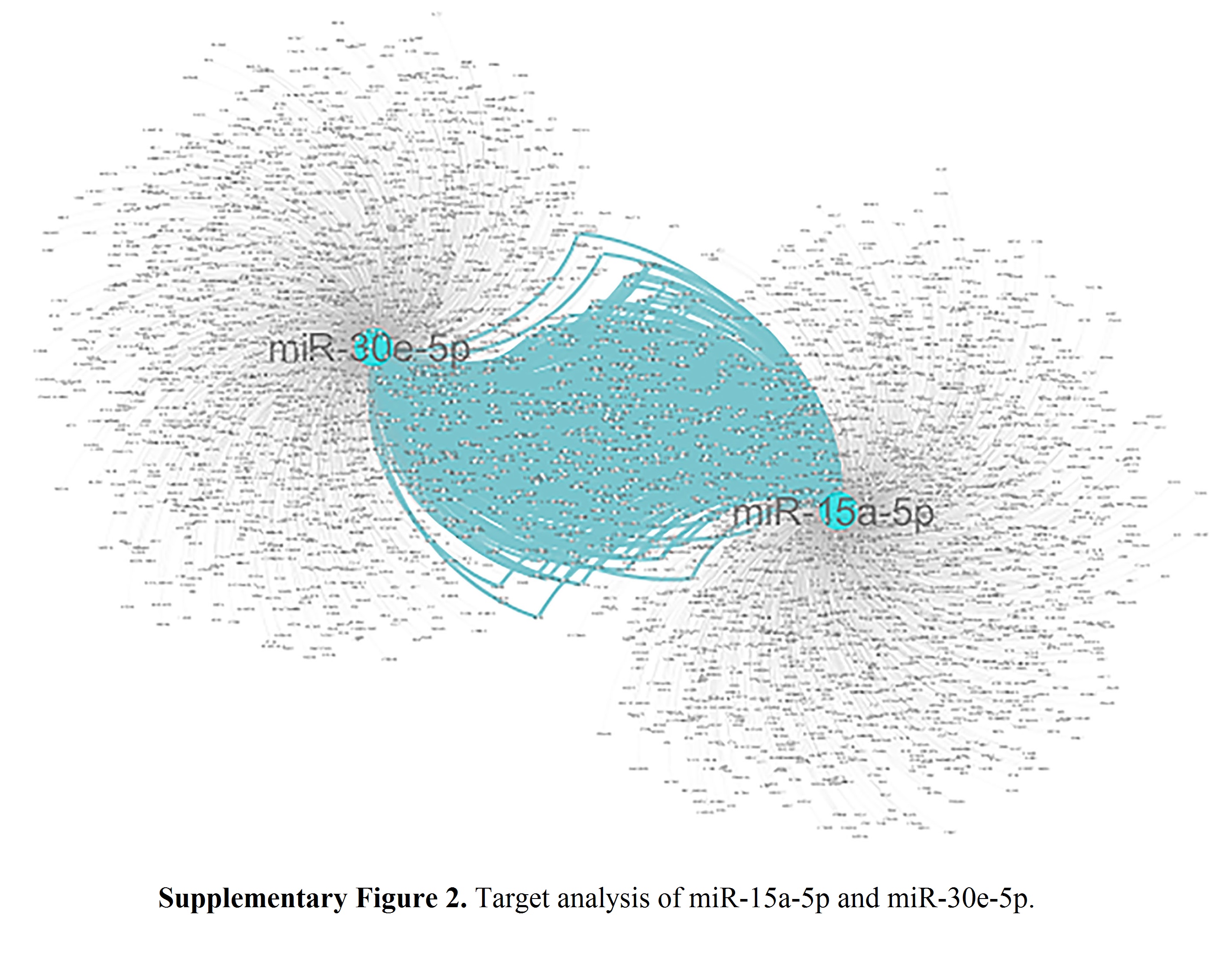

Supplement: Supplementary file 2 [file Image_2.JPEG]
